# Outstanding Reviewers for *RSC Chemical Biology* in 2023

**DOI:** 10.1039/d4cb90038c

**Published:** 2024-09-06

## Abstract

We would like to take this opportunity to thank all of *RSC Chemical Biology's* reviewers for helping to preserve quality and integrity in chemical science literature. We would also like to highlight the Outstanding Reviewers for *RSC Chemical Biology* in 2023.

## Abstract 2

We would like to take this opportunity to highlight the Outstanding Reviewers for *RSC Chemical Biology* in 2023, as selected by the editorial team for their significant contribution to the journal.

## Graphical abstract

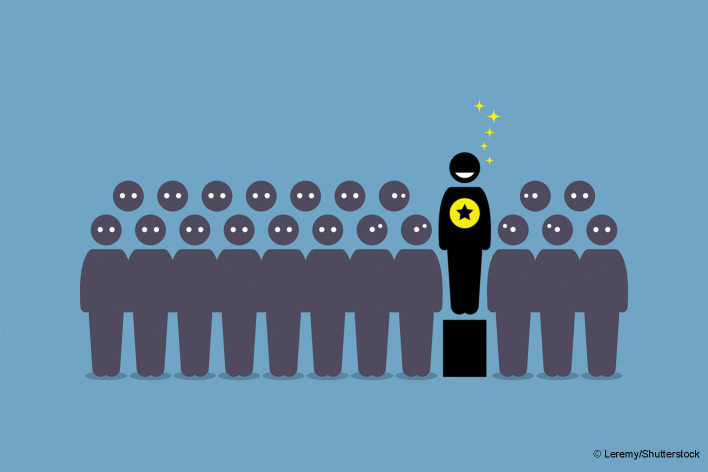

Each one of our outstanding peer reviewers has been carefully selected by our editorial team and includes active researchers who have made significant contributions to peer review and have gone above and beyond in their actions. Our Outstanding Reviewers from 2023 have been chosen based on several different measures, including the number, timeliness and quality of the reports completed over the last 12 months. In addition to reviewers who provided a high number of quality reports, we are pleased to highlight reviewers who provided exceptionally thorough and detailed reports and reviewers who made additional efforts to aid authors in improving their manuscripts.

 

Professor Anne C. Conibear

Vienna University of Technology

ORCID: 0000-0002-5482-6225

 

Dr Malte Gersch

Technische Universität Dortmund

ORCID: 0000-0003-2767-9589

 

Dr Jason Holland

Universität Zürich

ORCID: 0000-0002-0066-219X

 

Professor Andrew G. Jamieson

University of Glasgow

ORCID: 0000-0003-1726-7353

 

Dr Gerbrand van der Heden van Noort

Leiden University Medical Centre

ORCID: 0000-0001-5955-6431

 

Dr Gerd Wagner

Queen's University Belfast

ORCID: 0000-0003-1086-2301

 

Dr Lin Yuan

Hunan University

ORCID: 0000-0002-1015-5319

 

Dr Guangyu Zhu

City University of Hong Kong

ORCID: 0000-0002-4710-7070

 

Dr Taotao Zou

Sun Yat-Sen University

ORCID: 0000-0001-9129-4398

 

We would also like to thank the *RSC Chemical Biology* Editorial Board and Advisory Board and the chemical biology community for their continued support of the journal, as authors, reviewers and readers.

We continue to work on improving the diversity of our reviewer pool to reflect the diversity of the communities that we serve.

 

Dr Anna Rulka, Executive Editor

Dr Viktoria Titmus, Editorial Manager

